# SCOPe: classification of large macromolecular structures in the structural classification of proteins—extended database

**DOI:** 10.1093/nar/gky1134

**Published:** 2018-11-30

**Authors:** John-Marc Chandonia, Naomi K Fox, Steven E Brenner

**Affiliations:** 1 Environmental Genomics and Systems Biology Division, Lawrence Berkeley National Laboratory, Berkeley, CA 94720, USA; 2 Molecular Biophysics and Integrated Bioimaging Division, Lawrence Berkeley National Laboratory, Berkeley, CA 94720, USA; 3 Department of Plant and Microbial Biology, University of California, Berkeley, CA 94720, USA

## Abstract

The SCOPe (Structural Classification of Proteins—extended, https://scop.berkeley.edu) database hierarchically classifies domains from the majority of proteins of known structure according to their structural and evolutionary relationships. SCOPe also incorporates and updates the ASTRAL compendium, which provides multiple databases and tools to aid in the analysis of the sequences and structures of proteins classified in SCOPe. Protein structures are classified using a combination of manual curation and highly precise automated methods. In the current release of SCOPe, 2.07, we have focused our manual curation efforts on larger protein structures, including the spliceosome, proteasome and RNA polymerase I, as well as many other Pfam families that had not previously been classified. Domains from these large protein complexes are distinctive in several ways: novel non-globular folds are more common, and domains from previously observed protein families often have N- or C-terminal extensions that were disordered or not present in previous structures. The current monthly release update, SCOPe 2.07–2018-10–18, classifies 90 992 PDB entries (about two thirds of PDB entries).

## BACKGROUND

The Structural Classification of Proteins (SCOP) database (1–4) is a manually curated resource that organizes domains from proteins of known structure in a hierarchy according to their structural and evolutionary relationships (reference 5 describes the principles behind SCOP, and how to use it). Work on the SCOP version 1 series concluded in 2009 with the release of SCOP 1.75. To continue its development, we created the SCOPe (SCOP—extended) database, which provides ongoing updates to the hierarchy and classification of new protein structures (6,7) from the Protein Data Bank (PDB) (8,9). The relationship of SCOPe to other structural classifications including SCOP2 (10), CATH (11), and ECOD (12) is discussed in detail elsewhere (7,13).

SCOPe organizes domains into the following hierarchical key levels: a *Family* contains related proteins with similar sequences but typically distinct functions. The *Superfamily* level brings together protein families with common functional and structural features inferred to share a common ancestor. Near the root, the basis of classification is purely structural: similar superfamilies without compelling evidence of a common evolutionary origin are grouped into *Folds*, which are arranged into *Classes* based mainly on secondary structure content and organization (14).

To facilitate computational analyses of the SCOPe hierarchy, we provide sequences and coordinate files for all SCOPe domains, as well as sequences for all PDB chains that are classified in SCOPe. Chemically modified amino acids are translated back to the original sequence, and sequences are curated to eliminate any errors resulting from automated parsing of PDB files. Because the majority of sequences in the PDB are very similar to others, SCOPe provides representative subsets of proteins that span all classified protein structures or domains while alleviating bias toward well-studied proteins. The highest quality representative in each subset is chosen using AEROSPACI scores (15), which provide a numeric estimate of the quality and precision of crystal structures. These resources were previously released as a distinct database, the ASTRAL compendium (15–17); however, newer releases of ASTRAL are fully integrated into SCOPe. These data may be downloaded in parseable files, or in a SQL database. Our SQL database contains additional information not currently available in parseable files, such as metadata extracted from PDB entries, including cross-references to other databases such as UniProt (18)

We produce a stable release of SCOPe (and all associated ASTRAL resources) roughly once each year, in which the hierarchy itself is updated by manual curation, primarily to add new superfamilies. Manual curation of superfamilies is a key feature of SCOPe, in which proteins with similar three-dimensional structure and no recognizable sequence similarity are examined by an expert curator to determine if they possess structural and functional features indicative of homology. If convincing evidence is found of an evolutionary relationship, this is annotated in SCOPe by grouping the homologous domains into a single superfamily; if the evidence is not compelling, the domains are annotated as having a common fold but not grouped into a superfamily. Once at least one structure from each SCOPe family has been classified by a human expert, most other structures from that family are added automatically using our rigorously validated software pipeline (6). Because hundreds of new structures from each weekly release of the PDB are classified by this pipeline, we release periodic SCOPe updates (approximately monthly) to supplement the stable releases.

In addition to adding new superfamilies, manual curation in each stable SCOPe release can also involve other changes to the hierarchy. If two formerly distinct superfamilies are discovered to be related, for example on the basis of a newly discovered structure of an evolutionary intermediate, our curator would merge the two superfamilies into one. Manual curation is also used to make changes to domain boundaries; for example, a single domain may be split into multiple domains if different parts of the domain are discovered in different evolutionary contexts (5,7). Finally, manual curation is used to correct errors discovered in previous releases: although the error rate for manually classified entries in SCOP is an exceptionally low 0.08% (6), errors are occasionally identified and corrected in the subsequent stable release. Examples of all types of curation have been previously discussed (6,7). All stable releases of SCOP and SCOPe to date are summarized in Table 1.

**Table 1.** SCOP growth

Release	Freeze date	Release date	Months to release	Total PDB entries	Total PDB entries classified	Total domains classified
SCOP 1.55	2001–03	2001–07	4	13 307	13 228	31 474
SCOP 1.57	2001–10	2002–01	3	14 833	14 736	35 755
SCOP 1.59	2002–03	2002–05	2	16 067	15 985	39 893
SCOP 1.61	2002–09	2002–11	2	17 510	17 411	44 327
SCOP 1.63	2003–03	2003–06	3	19 049	18 951	49 497
SCOP 1.65	2003–08	2003–12	4	20 715	20 619	54 745
SCOP 1.67	2004–05	2005–02	9	24 151	24 036	65 122
SCOP 1.69	2004–10	2005–07	9	26 124	25 972	70 859
SCOP 1.71	2005–01	2006–10	21	27 844	27 599	75 930
SCOP 1.73	2007–09	2007–11	2	44 156	34 494	97 178
SCOP 1.75	2009–02	2009–06	4	53 832	38 221	110 800
SCOPe 2.01	2012–02	2012–03	1	76 312	49 219	135 634
SCOPe 2.02	2012–11	2013–01	2	83 296	49 560	136 313
SCOPe 2.03	2013–08	2013–10	2	90 354	59 514	167 547
SCOPe 2.04	2014–04	2014–07	3	96 087	67 580	192 710
SCOPe 2.05	2014–12	2015–02	3	102 263	71 015	203 026
SCOPe 2.06	2016–01	2016–02	1	113 035	77 439	244 326
SCOPe 2.07	2017–12	2018–03	3	133 747	87 224	276 231

The number of entries and domains in each release of SCOP that used stable identifiers. For each release, the ‘freeze date,’ or date after which no new PDB entries were to be classified in the release, is given. In practice, some entries released just after the freeze date were sometimes included. The total number of PDB entries that contained protein structures, were not obsolete as of the freeze date, or which were included in each release, is given, as well as the number of PDB entries that were included in each release and the number of domains in these entries. These counts differ slightly from the counts in (6) due to corrections to the dates on which some entries became obsolete. Release 1.71 was the most recent comprehensive SCOP release (i.e. one in which nearly all PDB entries available prior to the freeze date were classified).

## MANUAL CURATION PRIORITIES

Based on a study of 571 recent articles that cited SCOP (13), we found that one large category of users are researchers who use SCOPe as a ‘gold standard’ for benchmarking computational algorithms, or to create training sets to aid in setting algorithmic parameters. Having SCOPe classify newly structurally characterized proteins, and especially new protein families, is critical for these users to improve the scientific validity of studies derived from SCOPe data.

We prioritized manual curation of new structures by focusing on those Pfam (19,20) families with the largest number of structures, but without any structure classified in SCOP or SCOPe. This prioritization reflects the hypothesis that protein families classified in Pfam are likely to be of more interest to the biological community than proteins not in Pfam, as Pfam is human curated. We prioritized unclassified Pfam sequence families with the most three-dimensional structures characterized, because larger numbers of structures may reflect a greater degree of scientific interest, and because once one structure is manually classified, it may be used as a model for classifying other structures in the family. As we previously reported, though more than 3000 Pfam families with known structures are not currently classified in SCOPe (7), the majority of these have only a single structure. As expected, the relationship between Pfam families and SCOPe families (or superfamilies) is not 1 to 1 (21); instead, we find that approximately half of newly classified Pfam families correspond to a new SCOPe fold or superfamily, while classification of the others identifies previously unannotated remote homologs within superfamilies (7). In about half of the latter cases, these relationships of remote homology have been previously annotated in Pfam by grouping multiple Pfam families into a single clan (22). When other members of a Pfam clan have previously been classified in SCOPe, the newly classified Pfam family often corresponds to a new family within the same SCOPe superfamily as the other clan members.

Recent advances in high resolution cryo-electron microscopy have contributed a wealth of novel structures (23). Many of these structures are of large macromolecular complexes. Large structures (whether determined by cryo-EM or by traditional methods) are usually of great interest to classify in SCOPe: because each structure contains multiple proteins, they often contain at least one protein from a family that had not previously been structurally characterized. Many such proteins are non-globular, and therefore unable to fold (or sufficiently flexible to make physiological structure determination infeasible) without the other proteins in the complex. In addition to these non-globular folds, non-compact regions of previously characterized folds are often seen in these structures. Such regions may have been disordered in prior structures, or proteolytically cleaved in order to determine the structure. Finally, these structures often contain ‘domain swaps’ in which structural elements from adjacent domains switch places with similar elements from an adjacent structure (24).

## FINDINGS FROM LARGE STRUCTURES

In SCOPe 2.07, we classified cryo-EM structures of several spliceosome complexes: the first structure of the yeast spliceosome (PDB entry: 3jb9; 25), the U4/U6.U5 tri-snRNP spliceosome (PDB entry: 5gan; 26), the C complex spliceosome (PDB entry: 5gmk; 27), the activated spliceosome (PDB entry: 5lqw; 28) and the B complex spliceosome (PDB entry: 5o9z; 29). We also classified crystal structures of the 26S proteasome (PDB entry: 4cr2; 30) and of RNA polymerase I (PDB entry: 4c3h; 31). Some findings from these structures are discussed below.

### Non-globular folds

**Figure 1.**
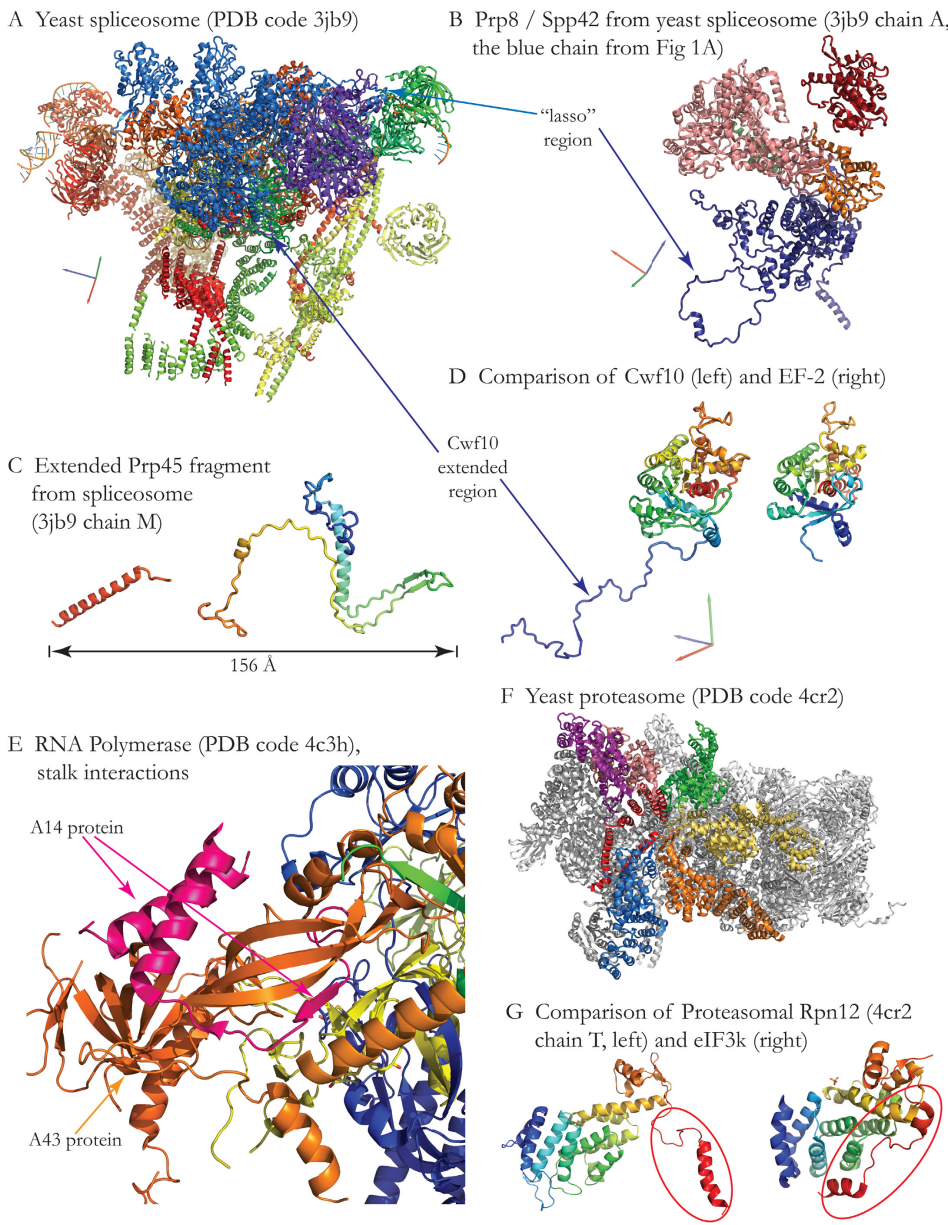
(**A**) The spliceosome complex 3jb9 is oriented with the two largest subunits at the top. Prp8 is in blue. Cwf10 is in purple. Note two interactions between the domains: on the right side of the figure, a loop of Prp8 (blue) forms a ‘lasso’ around part of Cwf10. Toward the bottom of the area between the two domains, a long extended region of Cwf10 (purple) interacts with Prp8 and other nearby subunits. Axes (x, y and z colored red, green and blue, respectively) indicate the orientation of (B) and (D) relative to (A). **(B)** Spliceosome component Prp8 contains five domains, four of which are homologous to domains previously seen in other proteins. The large, mainly α-helical N-terminal domain shown in dark blue has never before been structurally characterized. It includes the ‘lasso’ region (bottom left) that binds Cwf10, as well as an extended helix (bottom right) that binds to another subunit, Cwf14. The second domain is a bromodomain shown in green. The third domain (pink) is homologous to retroviral reverse transcriptase domains. The fourth domain (orange) is homologous to restriction endonucleases. The latter three domains had been predicted bioinformatically (33), and are strongly supported by the structure; all three represented new families in SCOPe, in existing superfamilies. The fifth domain (red) is ribonuclease H-like, as previously classified in SCOP. (**C**) The extended Prp45 fragment from the yeast spliceosome structure comprises about half of the full-length protein, and binds to at least nine other subunits (25). The observed parts of the structure span a distance of over 150 Å. **(D)** The first domain in spliceosome component Cwf10 (compact portion, left) belongs to a previously structurally characterized family of proteins that includes Elongation Factor 2 (EF-2). A typical structure of EF-2 (d3b8he1, right) is shown for comparison in the same orientation. The extended region of Cwf10 is stabilized by interactions with Prp8. (**E**) RNA Polymerase I. A close-up view of the interactions between the A14 protein and the A43 protein in the RNA Polymerase I stalk is shown. A14 is shown in pink, and A43 is in orange. A14 forms a novel non-globular fold consisting of an α hairpin and several β strands involved in heteromeric binding with A43. (**F**) The 26S proteasome complex 4cr2 is oriented with the regulatory Proteasome-COP9-Initiation factor 3 (PCI) ring on the front left. The horseshoe-shaped ring comprises 6 homologous subunits, Rpn3/5/6/7/9/12, shown in pink/orange/yellow/green/blue/purple. The C-terminal helix of each domain is colored red. (**G**) One of the PCI subunits, Rpn12, is shown on the left. It contains two domains, a TPR-like superhelical domain on the left side of the structure and a ‘winged helix’ domain, top right of the structure. The C-terminal helix (circled in red) is part of the ‘winged helix’ domain. A previously characterized homolog, eIF3k, is shown on the right. eIF3k contains the same two domains, but the C-terminal helix (circled in red) is broken and packed against the N-terminal domain.

Figure 1A shows a view of the spliceosome complex (PDB entry: 3jb9), oriented with the two largest proteins in the complex on top. Prp8, in blue, is the major component of the spliceosome’s catalytic core. Cwf10, in purple, is a GTPase that is homologous to Elongation Factor 2 (EF-2). Prp8 contains a lasso-like region that wraps around part of Cwf10. Visualized on its own (Figure 1B, which is rotated relative to Figure 1A to provide a clear view of all domains), Prp8 has five domains, four of which are homologous to domains observed previously in other structures. However, the first domain of Prp8 (shown in dark blue in Figure 1B) was not observed in earlier structures of Prp8 that were determined in isolation (25). The overall fold of the domain is not similar to any previously observed: in addition to a fairly compact helical region, it also comprises several extended non-globular regions, including the lasso and an N-terminal helix. All five of the spliceosome structures that were manually classified in SCOPe 2.07 include similar non-globular domains that largely consist of extended helices and loops, and are therefore unlikely to be stable in the absence of interacting partners. A striking example is Prp45 (Figure 1C), which forms a scaffold that interacts with nine other subunits (not shown, as they surround Prp45 on all sides), spanning an extended region more than 150 Å across.

Non-globular folds have been observed to be abundant in ribosomal complexes (32), so it is not surprising that they are common in other large heteromeric complexes as well. Extended, non-globular chains such as Prp45 are somewhat challenging to classify in SCOPe, as there is not currently an explicit ‘non-globular’ class. Because the structurally characterized regions of Prp45 comprise only about half of the protein, we placed it in the ‘j: Peptides’ class, which contains fragments of longer proteins as well as short peptides. However, other databases such as the SCOP2 prototype (10) create a specific category for longer, non-globular proteins and larger fragments of these proteins, and it is likely that we will need to add such a category to future SCOPe releases.

### Non-globular regions of globular folds

The spliceosome structure also contains a striking example of a non-globular extension of a previously characterized globular fold. Figure 1D compares the first domain of the spliceosomal GTPase Cwf10 (left structure in 1D; also purple chain in Figure 1A) to the structure of a homolog, elongation factor 2 (EF-2, right). The Cwf10 domain includes a long extended region (purple) that is stabilized by interactions with Prp8. Following the principles of classification from the SCOP version 1 series, we consider this region to be an extension of the common fold of these homologous domains, rather than a separate domain. If a homolog of this region is ever observed independently in other proteins, we would split the region off into a separate domain.

The RNA Polymerase I structure (Figure 1E) provides another example of a fold that is partly non-globular. In RNA Polymerase I, proteins A43 and A14 form a heterodimeric stalk that provides a platform for initiation factors and interacts with newly synthesized RNA (31). A43 is homologous to RpoE, a ribosome-binding factor that was previously classified in SCOP. A14 forms a novel fold consisting of an α-helical hairpin, and a β strand that becomes part of the β sheet at the core of A43. A14 is homologous to a domain of RpoF, another ribosome-binding factor that is classified in SCOP; however, none of the RpoF structures solved to date included the homologous domain. Therefore, A14 was classified in a new fold in the ‘g: Small proteins’ SCOPe class, and any future structures of RpoF that include the domain homologous to A14 will be divided appropriately.

### Domain swaps

In the 26S proteasome structure (Figure 1F), six homologous heteromeric subunits form the PCI subcomplex, a horseshoe-shaped structure shown in color. Each of the subunits comprises two domains: an α-helical superhelix from the ‘TPR-like’ family, named due to homology to tetratricopeptide repeats (TPR), and a ‘winged helix’ domain. ‘Winged helix’ domains contain three helices and a small β sheet, and often bind DNA. Some members of the superfamily contain an additional N-terminal α helix. In eukaryotic translation initiation factor 3 subunit 12 (eIF3k), this α helix packs against the TPR-like domain (PDB entry: 1rz4; 36). However, in all six subunits of the PCI complex, the N-terminal helices (colored red in Figure 1F) are longer and pack against each other and against nearby TPR-like domains, rather than folding back against the TPR-like domain from the same chain. Figure 1G compares the structure of eIF3k with Rpn12, one of the PCI complex subunits. The PCI subunits exhibit a type of ‘domain swapping’ in which a structural element (the N-terminal helix of each subunit, circled in red) contacts the same type of structure (helical bundles) that it contacts in monomeric structures, but in the multimeric complex the contacts are all with nearby domains instead.

### New SCOPe folds, superfamilies and families

The first structure of the yeast spliceosome (25) included proteins classified into 16 different Pfam families that had not previously been classified in SCOPe. Classification of this spliceosome structure resulted in six new folds, three new superfamilies in previously annotated folds and five new families in previously annotated superfamilies, as well as 21 other domains in previously annotated families. We classified at least one example of every distinct protein in the structure, which includes some regions not classified in Pfam. The correspondence between Pfam annotations and SCOPe domains is complex, as illustrated by the following example of the Prp8 protein, the major component of the spliceosome’s catalytic core. In contrast to the five SCOPe domains discussed above and in Figure 1B, Pfam annotates eight families in Prp8, one of which is a motif. The novel N-terminal fold in Prp8 spans one Pfam family (PF08082, PRO8NT) and half of another (PF08083, PROCN) as well as many residues not classified by Pfam. The second SCOPe domain (a bromodomain) corresponds to the other half of the PF08083 family. The bromodomain is not hit by the Pfam bromodomain model (PF00439), nor are PF00439 and PF08083 in a clan.

In addition to the newly characterized folds and superfamilies (discussed above), the cases where we identified new families in existing superfamilies are also quite interesting, as in each case the spliceosome structure confirmed remote homology to other proteins that was not readily apparent from sequence alone. Remarkably, for three domains in Prp8 (Figure 1B), remote homology to other proteins had been predicted by a sophisticated bioinformatic analysis (33), and we found all three predictions to be well-supported by the structure. We annotated this finding in SCOPe by placing all three domains in new families within existing superfamilies.

Each of the other macromolecular structures we characterized yielded a wealth of new SCOPe folds, superfamilies and families as well. The four additional spliceosome complexes include an additional 19 Pfam families that had not been previously classified in SCOPe. After classification in SCOPe, these structures yielded 10 new folds, 1 new superfamily in an existing fold and 1 new family in an existing superfamily. Although some individual proteasome subunits had previously been structurally determined in isolation and classified in SCOP, the crystal structure of the entire 26S proteasome (30) yielded structures for an additional five Pfam families that had not been previously classified. Classification of these structures resulted in one new fold, three new superfamilies in existing folds and two new families in existing superfamilies. Similarly, although all subunits of RNA Polymerase II had been previously classified in SCOP, the crystal structure of RNA Polymerase I (31) provided the first structural characterization of an additional four Pfam families, which include proteins that are specific to the latter complex. Classification of these structures resulted in one new fold and two new families within existing superfamilies.

## IMPLICATIONS OF INCREASING NUMBERS OF MACROMOLECULAR COMPLEXES

As cryo-EM is used to solve the structures of more macromolecular complexes, we anticipate that more new superfamilies from non-globular folds will be structurally characterized and added to SCOPe, and currently classified superfamilies will expand to contain a more diverse set of structures than they currently do. The increased structural heterogeneity may pose challenges for bioinformatic analyses of the data, especially those methods that depend on structural alignments. We expect to discover more examples of domain swapping, as the greater number of inter-domain contacts will lead to more chances for this to happen, as we observed in the large complexes discussed here.

More structural heterogeneity will also pose some challenges for automatic identification of domains. Although the current algorithm for automated domain classification is sequence-based and does not rely on information from structural superposition (7), we anticipate adding methods based on structural comparison to future versions of the algorithm in order to increase the fraction of newly solved structures that can be automatically classified. As with our current method for automated classification, new methods will be carefully benchmarked against prior SCOP and SCOPe releases to maintain the high accuracy found in fully manually curated SCOP releases (6,7). A silver lining is that even in a structurally heterogeneous superfamily, one only needs a good match to a single manually classified structure in order to automatically classify homologs.

## PROSPECTS FOR FULL CLASSIFICATION OF THE PDB

As shown in Table 1, all releases of SCOP through version 1.71 were comprehensive, classifying virtually every entry available in the PDB on the freeze date. As the PDB’s growth rate increased, building new comprehensive versions of SCOP required increasing amounts of curation time to classify all structures available on a particular date. Automated classification methods were introduced in SCOP 1.73 to abet manual curation, but later releases were not comprehensive (6). Comprehensive coverage is necessary in order to perform analyses of the full repertoire of protein structures (34,35), without being biased by the interests of database curators.

Although manual curation was re-introduced to SCOPe in version 2.04 (7), the project is currently being maintained without funding, so time available for expert manual curation of new releases is limited. Nevertheless, the combination of limited manual curation and precise automated classification has enabled us to keep pace with the growth of the PDB. As shown in Table 1, the seven stable SCOPe releases to date have consistently classified about two thirds of protein structures in the PDB at the time of each release, as the PDB has roughly doubled in size. We anticipate that a focused curation effort, if funded, would enable us to clear the backlog of thousands of structurally characterized but unclassified Pfam families and allow us to return to near-complete classification of all PDB entries (7).
